# Supplementary material for: Rapid and repeated evolution of myosin copy number in threespine stickleback
Source: bioRxiv. 2025 Dec 25:2025.12.22.696110. Preprint. [Version 1] doi: 10.64898/2025.12.22.696110 (PMC12776057; doi:10.64898/2025.12.22.696110)
Supplement: Supplement 2 [file NIHPP2025.12.22.696110v1-supplement-2.pdf]

bioRxiv preprint doi: https://doi.org/10.64898/2025.12.22.696110; this version posted December 25, 2025. The copyright holder for this preprint (which was not certified by peer review) is the author/funder, who has granted bioRxiv a license to display the preprint in perpetuity. It is made available under aCC-BY 4.0 International license.

Supplemental information

Table S1. Global stickleback samples from Roberts Kingman et al.[26] used for *C3* read depth calculations, related to Figures 1, S1, and S2

Table S2. Samples used for generating *MYH3C* assemblies, related to Figure 2

bioRxiv preprint doi: https://doi.org/10.64898/2025.12.22.696110; this version posted December 25, 2025. The copyright holder for this preprint (which was not certified by peer review) is the author/funder, who has granted bioRxiv a license to display the preprint in perpetuity. It is made available under aCC-BY 4.0 International license.

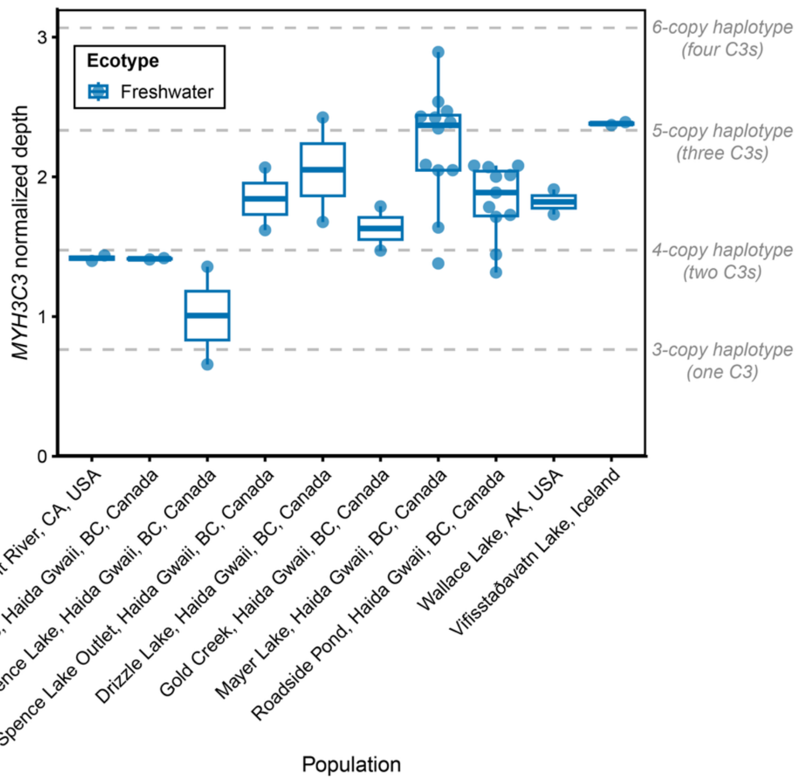

Figure S1. Within-population variation in *MYH3C3* copy number

MYH3C3 read depth normalized by mean autosomal read depth determined for female stickleback from Roberts Kingman et al.[26] in populations represented by at least two individual fish. Roadside Pond was derived from a transplant of Mayer Lake fish in 1993[117]. Read depth differences support variation in *MYH3C3* copy number within Drizzle Lake, Spence Lake, Mayer Lake, and Roadside Pond. Dashed lines indicate read depth calculated from simulated reads of different assembled 3- to 6-copy myosin haplotypes (one to four *MYH3C3* copies per haplotype; Figure 2). See Table S1 and Roberts Kingman et al.[26] for more information about each sample.

bioRxiv preprint doi: https://doi.org/10.64898/2025.12.22.696110; this version posted December 25, 2025. The copyright holder for this preprint (which was not certified by peer review) is the author/funder, who has granted bioRxiv a license to display the preprint in perpetuity. It is made available under aCC-BY 4.0 International license.

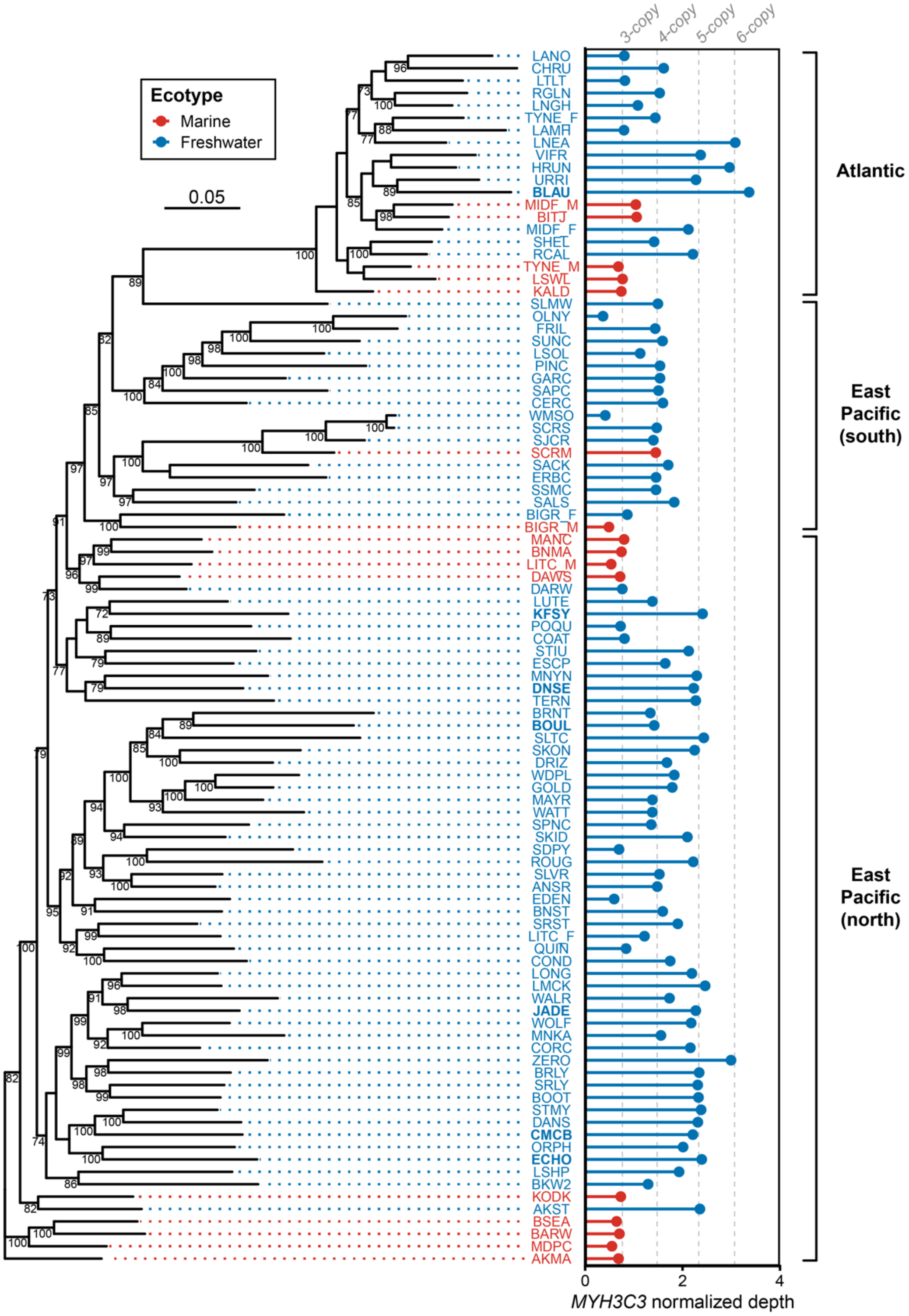

Figure S2. Repeated evolution of *MYH3C3* copy number across global stickleback populations

Phylogenetic tree based on 100,000 genome-wide neutral SNPs from the 15 marine (red) and 81 freshwater (blue) stickleback from Figure 2B. Bootstrap values of ≥70 are labeled below each node. Branch lengths are based on the number of inferred substitutions, indicated by the scale bar. Normalized

bioRxiv preprint doi: https://doi.org/10.64898/2025.12.22.696110; this version posted December 25, 2025. The copyright holder for this preprint (which was not certified by peer review) is the author/funder, who has granted bioRxiv a license to display the preprint in perpetuity. It is made available under aCC-BY 4.0 International license.

MYH3C3 read depth is plotted for each individual as previously described in Figure 2B. Freshwater populations with higher *MYH3C3* read depth are interspersed with marine populations with lower *MYH3C3* read depth in both the Pacific and Atlantic basins, suggesting *MYH3C3* copy number expansions have likely evolved multiple times.

bioRxiv preprint doi: https://doi.org/10.64898/2025.12.22.696110; this version posted December 25, 2025. The copyright holder for this preprint (which was not certified by peer review) is the author/funder, who has granted bioRxiv a license to display the preprint in perpetuity. It is made available under aCC-BY 4.0 International license.

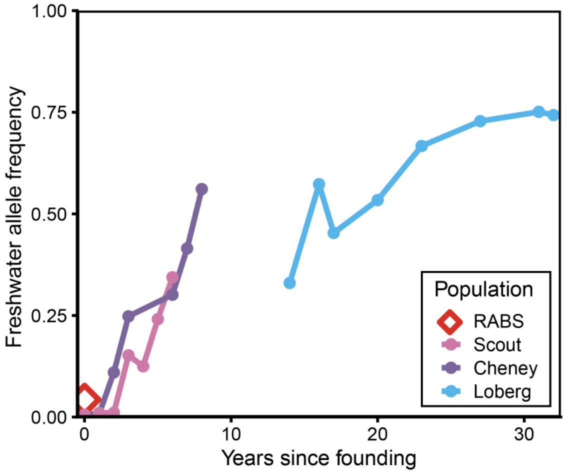

Figure S3. SNP allele frequencies associated with the freshwater *MYH3C* allele increase rapidly when marine fish are introduced to freshwater

Allele frequencies for the most significant SNP (ChrXIX:2,744,769 [*gasAcu1-4* reference]) within the Sensitive TempoPeak[26] overlapping the ChrXIX *MYH3C* locus. Allele frequencies of the freshwater allele are low in the founder marine population (RABS, red) but rise to over 30% frequency within several generations in freshwater habitats (Scout [pink], Cheney [purple], Loberg [light blue]).

bioRxiv preprint doi: https://doi.org/10.64898/2025.12.22.696110; this version posted December 25, 2025. The copyright holder for this preprint (which was not certified by peer review) is the author/funder, who has granted bioRxiv a license to display the preprint in perpetuity. It is made available under aCC-BY 4.0 International license.

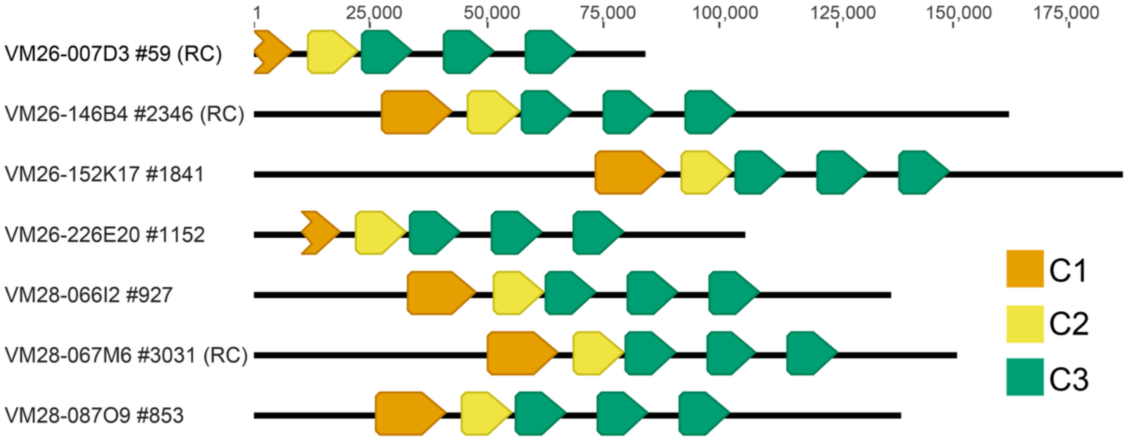

Figure S4. The BEPA reference stickleback has a 5-copy *MYH3C* haplotype

Representative individual Nanopore reads from all seven BACs that fully span duplicated *C3* copies derived from the female BEPA stickleback used to create the freshwater reference genome[25,38]. Individual DNA molecules were annotated with *C1* (orange), *C2* (yellow), and *C3* (green). All seven clones show three *C3* gene copies at the myosin locus, suggesting that the freshwater reference genome artificially collapsed *C3* copies during assembly. Some reads have been reverse complemented for visual clarity (RC). The reads from the VM26-007D3 and VM26-226E20 BAC clones only partially span *C1*, due the lengths of the longest *C3* spanning read (VM26-007D3) or to the size of the stickleback insert (VM26-226E20).

bioRxiv preprint doi: https://doi.org/10.64898/2025.12.22.696110; this version posted December 25, 2025. The copyright holder for this preprint (which was not certified by peer review) is the author/funder, who has granted bioRxiv a license to display the preprint in perpetuity. It is made available under aCC-BY 4.0 International license.

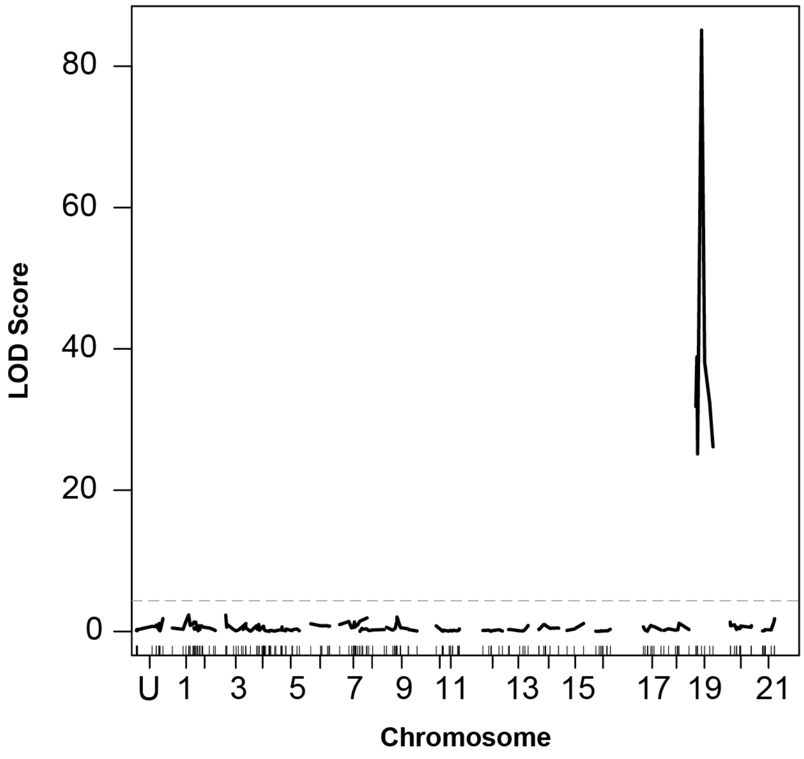

Figure S5. Myosin *C3* copy numbers map to a single major locus

C3 copy numbers were scored in 440 individual F2 progeny from a cross between marine (Bodega Bay, CA) and freshwater (Boulton Lake, BC, Canada) stickleback. A SNP marker adjacent to the *MYH3C* locus on ChrXIX explained nearly 60% of the variance in copy number (LOD = 85.1, PVE = 0.59, $p < 1x10^{-4}$), and no significant linkage was found to other chromosomes. The LOD score cutoff for a genome-wide α = 0.01 is shown with the dashed line. ChrU represents concatenated unassembled regions of the genome[25].

bioRxiv preprint doi: https://doi.org/10.64898/2025.12.22.696110; this version posted December 25, 2025. The copyright holder for this preprint (which was not certified by peer review) is the author/funder, who has granted bioRxiv a license to display the preprint in perpetuity. It is made available under aCC-BY 4.0 International license.

Table S3. Marine/freshwater divergent amino acids in each *MYH3C* copy.

Position	MYH3C copy	Marine aa	Freshwater aa	Marine aa type	Freshwater aa type	Same type?	Domain
57	C1	A	T	hydrophobic	polar	no	SH3
209	C1	T	A	polar	hydrophobic	no	Myosin motor
388	C1	M	L	hydrophobic	hydrophobic	yes	Myosin motor
468	C1	Y	F	hydrophobic	hydrophobic	yes	Myosin motor
470	C1	S	T	polar	polar	yes	Myosin motor
471	C1	M	L	hydrophobic	hydrophobic	yes	Myosin motor
507	C1	D	E	negative charge	negative charge	no	Myosin motor
571	C1	A	V	hydrophobic	hydrophobic	yes	Myosin motor
573	C1	A	G	hydrophobic	special	no	Myosin motor
728	C1	D	E	negative charge	negative charge	no	Myosin motor
1019	C1	A	S	hydrophobic	polar	no	Myosin tail
1058	C1	A	S	hydrophobic	polar	no	Myosin tail
1062	C1	I	V	hydrophobic	hydrophobic	yes	Myosin tail
1233	C1	G	A	special	hydrophobic	no	Myosin tail
1272	C1	H	Q	positive charge	polar	no	Myosin tail
1859	C1	R	K	positive charge	positive charge	yes	Myosin tail
29	C2	S	T	polar	polar	yes	
43	C2	A	V	hydrophobic	hydrophobic	yes	SH3
55	C3	K	R	positive charge	positive charge	yes	SH3
56	C3	E	D	negative charge	negative charge	no	SH3
76	C3	D	E	negative charge	negative charge	no	SH3
209	C3	T	A	polar	hydrophobic	no	Myosin motor
251	C3	S	G	polar	special	no	Myosin motor
257	C3	A	S	hydrophobic	polar	no	Myosin motor
325	C3	I	V	hydrophobic	hydrophobic	yes	Myosin motor
573	C3	G	A	special	hydrophobic	no	Myosin motor
589	C3	T	N	polar	polar	yes	Myosin motor
838	C3	T	S	polar	polar	yes	
885	C3	L	M	hydrophobic	hydrophobic	yes	Myosin tail
1019	C3	A	S	hydrophobic	polar	no	Myosin tail
1186	C3	S	A	polar	hydrophobic	no	Myosin tail
1244	C3	L	M	hydrophobic	hydrophobic	yes	Myosin tail
1365	C3	A	S	hydrophobic	polar	no	Myosin tail
1608	C3	L	M	hydrophobic	hydrophobic	yes	Myosin tail
1824	C3	A	T	hydrophobic	polar	no	Myosin tail
1929	C3	A	V	hydrophobic	hydrophobic	yes	

bioRxiv preprint doi: https://doi.org/10.64898/2025.12.22.696110; this version posted December 25, 2025. The copyright holder for this preprint (which was not certified by peer review) is the author/funder, who has granted bioRxiv a license to display the preprint in perpetuity. It is made available under aCC-BY 4.0 International license.

Table S4. *K*-mers used for RNA-seq analysis.

Set	*MYH3C* target	Allele target	Exon	*k*-mer (forward)	*k*-mer (reverse complement)
MCE1	*C1*	both	3	GCTATTTACCTTCGTAAGCCAGAGAGG	CCTCTCTGGCTTACGAAGGTAAATAGC
MCE1	*C2*	both	3	GCCATTTACCTTCGTAAGCCAGAGAAG	CTTCTCTGGCTTACGAAGGTAAATGGC
MCE1	*C3*	both	3	GCCATTTACCTTCGTAAGCCAGAGAGG	CCTCTCTGGCTTACGAAGGTAAATGGC
MCE2	*C1*	both	3	TGTACTTGAAGGCCACAATCCTCAAGA	TCTTGAGGATTGTGGCCTTCAAGTACA
MCE2	*C2*	both	3	TGTACTTGAAGGCCACAATCATCAAGA	TCTTGATGATTGTGGCCTTCAAGTACA
MCE2	*C3*	both	3	TGTATTTGAAGGCCAAAGTCATCAAGA	TCTTGATGACTTTGGCCTTCAAATACA
MCE3	*C1*	both	28	AGGACCAACTTAGCGAAGTGAAGACAA	TTGTCTTCACTTCGCTAAGTTGGTCCT
MCE3	*C2*	both	28	AGGACCAACTTAGCGAACTGAAGACAA	TTGTCTTCAGTTCGCTAAGTTGGTCCT
MCE3	*C3*	both	28	AGGACCAATTTAGCGAACTGAAGACAA	TTGTCTTCAGTTCGCTAAATTGGTCCT
MCE4	*C1*	both	33	GTGGAAACGGAGAAGGCTGAGATCCAG	CTGGATCTCAGCCTTCTCCGTTTCCAC
MCE4	*C2*	both	33	GTGGAAACAGAGAAGTCTGAGATCCAG	CTGGATCTCAGACTTCTCTGTTTCCAC
MCE4	*C3*	both	33	GTGGAAACAGAGAAGACTGAGATCCAG	CTGGATCTCAGTCTTCTCTGTTTCCAC
MCE5	*C1*	both	37	GAGCAAGACACGAGTGCTCACCTTGAG	CTCAAGGTGAGCACTCGTGTCTTGCTC
MCE5	*C2*	both	37	GAGCAAGACACTAGTGCTCACCTTGAG	CTCAAGGTGAGCACTAGTGTCTTGCTC
MCE5	*C3*	both	37	GAGCAAGACACGAGTTCTCACCTTGAG	CTCAAGGTGAGAACTCGTGTCTTGCTC
MCE6	*C1*	both	40	AATGCTCATCTGTCCAAATGCAGGAAG	CTTCCTGCATTTGGACAGATGAGCATT
MCE6	*C2*	both	40	AATGCTCATCTGTCCAAGTGCAGGAAG	CTTCCTGCACTTGGACAGATGAGCATT
MCE6	*C3*	both	40	AATACTCATCTGTCCAAGTGCAGAAAG	CTTTCTGCACTTGGACAGATGAGTATT
MCE7	*C1*	both	40	AACAAGCTGAGAGCAAAAACCCGTGAC	GTCACGGGTTTTTGCTCTCAGCTTGTT
MCE7	*C2*	both	40	AACAAGCTGAGAGCAAAAAGCCGTGAC	GTCACGGCTTTTTGCTCTCAGCTTGTT
MCE7	*C3*	both	40	AACAAGATGAGAGCAAAAAGTCGTGAC	GTCACGACTTTTTGCTCTCATCTTGTT
C1_ASE1	*C1*	BEPA	3	CCTCAAGAAAGAGACTGGCAAAGTCAC	GTGACTTTGCCAGTCTCTTTCTTGAGG
C1_ASE1	*C1*	RABS	3	CCTCAAGAAAGAGGCTGGCAAAGTCAC	GTGACTTTGCCAGCCTCTTTCTTGAGG
C1_ASE2	*C1*	BEPA	12	TTCTCAAGTGAAGAGAAGTTGAGCATC	GATGCTCAACTTCTCTTCACTTGAGAA
C1_ASE2	*C1*	RABS	12	TTCTCAAGTGAAGAGAAGCTGAGCATC	GATGCTCAGCTTCTCTTCACTTGAGAA
C1_ASE3	*C1*	BEPA	21	CACTGGTCACCATGACTCAGGCTTTGT	ACAAAGCCTGAGTCATGGTGACCAGTG
C1_ASE3	*C1*	RABS	21	CACTGGTCACAATGACTCAGGCTTTGT	ACAAAGCCTGAGTCATTGTGACCAGTG
C1_ASE4	*C1*	BEPA	27	GATGGAAATTGATGACCTCTCTAGCAA	TTGCTAGAGAGGTCATCAATTTCCATC
C1_ASE4	*C1*	RABS	27	GATGGAAATCGATGACCTCTCCAGCAA	TTGCTGGAGAGGTCATCGATTTCCATC
C1_ASE5	*C1*	BEPA	27	CCTCTCTAGCAACATGGAGGCTGTTGC	GCAACAGCCTCCATGTTGCTAGAGAGG
C1_ASE5	*C1*	RABS	27	CCTCTCCAGCAACATGGAGGGTGTTGC	GCAACACCCTCCATGTTGCTGGAGAGG
C1_ASE6	*C1*	BEPA	36	AGCTTGAGACTGACCTGGTCCAAGTCC	GGACTTGGACCAGGTCAGTCTCAAGCT

bioRxiv preprint doi: https://doi.org/10.64898/2025.12.22.696110; this version posted December 25, 2025. The copyright holder for this preprint (which was not certified by peer review) is the author/funder, who has granted bioRxiv a license to display the preprint in perpetuity. It is made available under aCC-BY 4.0 International license.

Set	*MYH3C* target	Allele target	Exon	*k*-mer (forward)	*k*-mer (reverse complement)
C1_ASE6	*C1*	RABS	36	AGCTCGAGACTGACCTGGTCCAG GTCC	GGACCTGGACCAGGTCAGTCTCG AGCT
C1_ASE7	*C1*	BEPA	38	AGCAAAGACGTGGAGCAGATGCC GTTA	TAACGGCATCTGCTCCACGTCTTT GCT
C1_ASE7	*C1*	RABS	38	AGCAGAGACGTGGAGCAGATGCC GTTA	TAACGGCATCTGCTCCACGTCTC TGCT
C1_ASE8	*C1*	BEPA	41	AGTGTATGTGTTAAGTCATATAAT ATG	CATATTATATGACTTAACACATAC ACT
C1_ASE8	*C1*	RABS	41	AGTGTATGTGTTAAATCATATAAT ATG	CATATTATATGATTTAACACATACA CT
C2_ASE1	*C2*	BEPA	3	AGGATTGAGGCCCAAACCGCACC ATTT	AAATGGTGCGGTTTGGGCCTCAA TCCT
C2_ASE1	*C2*	RABS	3	AGGATTGAGGCCCAAAGCGCACC ATTT	AAATGGTGCGCTTTGGGCCTCAA TCCT
C2_ASE2	*C2*	BEPA	5	CATATCTTCTCTGTCTCTGACAAC GCC	GGCGTTGTCAGAGACAGAGAAGA TATG
C2_ASE2	*C2*	RABS	5	CACATCTTCTCTGTCTCTGACAAC GCC	GGCGTTGTCAGAGACAGAGAAGA TGTG
C2_ASE3	*C2*	BEPA	13	TGCTTACCTGCTTGGTCTCAACTC TGC	GCAGAGTTGAGACCAAGCAGGTA AGCA
C2_ASE3	*C2*	RABS	13	TGCTTACCTGCTCGGTCTCAACTC TGC	GCAGAGTTGAGACCGAGCAGGTA AGCA
C2_ASE4	*C2*	BEPA	33	AAGCAGGTGGAAACAGAGAAGTC TGAG	CTCAGACTTCTCTGTTTCCACCTG CTT
C2_ASE4	*C2*	RABS	33	AAGCAAGTGGAAACAGAGAAGTC TGAG	CTCAGACTTCTCTGTTTCCACTTG CTT
C3_ASE1	*C3*	BEPA	3	GCCAAAGTCATCAAGAGAGATGG TGGC	GCCACCATCTCTCTTGATGACTTT GGC
C3_ASE1	*C3*	RABS	3	GCCAAAGTCATCAAGAAAGAGGG TGGC	GCCACCCTCTTTCTTGATGACTTT GGC
C3_ASE2	*C3*	BEPA	4	AGGACAGTTAAAGAAGAAGAAAT CTTT	AAAGATTTCTTCTTCTTTAACTGTC CT
C3_ASE2	*C3*	RABS	4	AGGACAGTTAAAGAAGATGAAATC TTT	AAAGATTTCATCTTCTTTAACTGT CCT
C3_ASE3	*C3*	BEPA	16	CCTACCAAGGGCAAGGCTGAGGC CCAC	GTGGGCCTCAGCCTTGCCCTTGG TAGG
C3_ASE3	*C3*	RABS	16	CCTACCAAGGGCAAGGCTGAGGG CCAC	GTGGCCCTCAGCCTTGCCCTTGG TAGG
C3_ASE4	*C3*	BEPA	21	CACTGGTCACGATGACCCAGGCT TTGT	ACAAAGCCTGGGTCATCGTGACC AGTG
C3_ASE4	*C3*	RABS	21	CACTGGTCACGATGACTCAGGCT TTGT	ACAAAGCCTGAGTCATCGTGACC AGTG
C3_ASE5	*C3*	BEPA	28	GATGTGCCGTACTCTTGAGGACC AATT	AATTGGTCCTCAAGAGTACGGCA CATC
C3_ASE5	*C3*	RABS	28	GCTGTGCCGTACTCTTGAGGACC AATT	AATTGGTCCTCAAGAGTACGGCA CAGC
C3_ASE6	*C3*	BEPA	30	AAACGGTGAGGTGTCTCAGTGGA GATC	GATCTCCACTGAGACACCTCACC GTTT
C3_ASE6	*C3*	RABS	30	AAACGGTGAGGTGGCTCAGTGGA GATC	GATCTCCACTGAGCCACCTCACC GTTT
C3_ASE7	*C3*	BEPA	38	GAGACTGAGCAGAGACGTGGAGT GGAC	GTCCACTCCACGTCTCTGCTCAG TCTC
C3_ASE7	*C3*	RABS	38	GAGGCTGAGCAGAGACGTGGAGT GGAC	GTCCACTCCACGTCTCTGCTCAG CCTC
C3_ASE8	*C3*	BEPA	38	GTGGACGCCGTCAAAGGTGTTCG CAAA	TTTGCGAACACCTTTGACGGCGT CCAC
C3_ASE8	*C3*	RABS	38	GTGGACGCCGTCAAGGGTGTTCG CAAA	TTTGCGAACACCCTTGACGGCGT CCAC
C3_ASE9	*C3*	BEPA	40	ATTGCTGAGTCTCAAGTCAACAAG ATG	CATCTTGTTGACTTGAGACTCAGC AAT
C3_ASE9	*C3*	RABS	40	ATTGCTGAGTCTCAGGTCAACAA GATG	CATCTTGTTGACCTGAGACTCAG CAAT

bioRxiv preprint doi: https://doi.org/10.64898/2025.12.22.696110; this version posted December 25, 2025. The copyright holder for this preprint (which was not certified by peer review) is the author/funder, who has granted bioRxiv a license to display the preprint in perpetuity. It is made available under aCC-BY 4.0 International license.

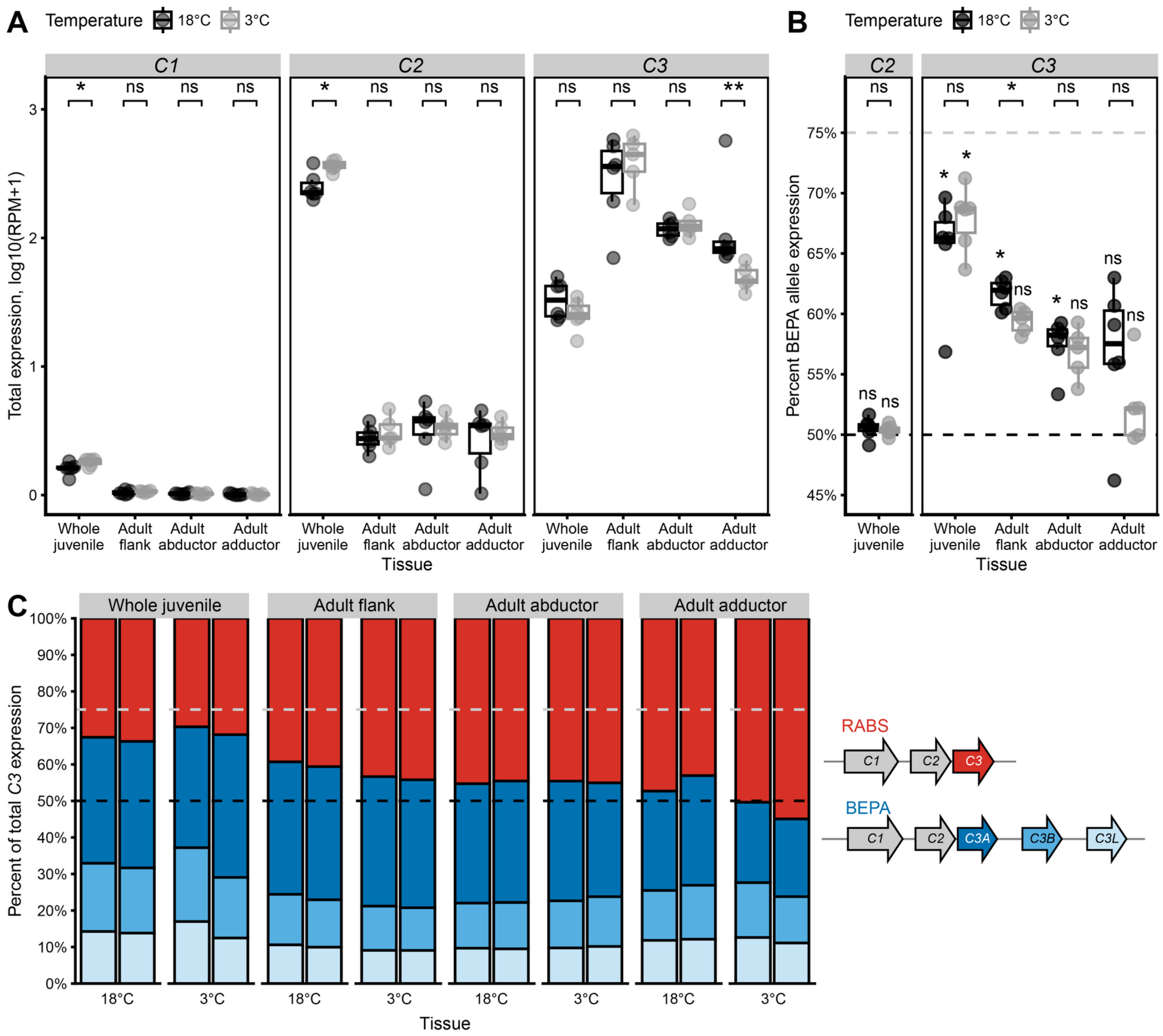

Figure S6. ***MYH3C*** **expression at different temperatures**

(A) Box plots showing the relative expression levels of *MYH3C* copies in RABS x BEPA F1 hybrid females based on seven sets of MCE 27-mers. Fish were acclimated to either standard 18°C (black) or cold 3°C (grey) temperatures. For all conditions, *n* = 6, except for 3°C adult tissues where *n* = 5. Gene expression differences under each temperature condition were compared using a two-sided Wilcoxon rank sum test. Expression of *C1* and *C2* increases at 3°C in juveniles while expression of *C3* decreases at 3°C in adult adductor muscle.

(B) Box plots showing allele-specific expression (ASE) of the BEPA vs. RABS alleles based on ASE *k*-mer sets (four *C2* sets and nine *C3* sets) for each *MYH3C* copy under 18°C or 3°C conditions. Deviation from an equal ratio of expression from the BEPA and RABS alleles (50% BEPA allele expression, black dashed line) was evaluated using a one-sample Wilcoxon rank sum test (mu = 0.5; shown directly above each box plot). The BEPA allele expression would be 75% with expression proportional to *C3* copy number (grey dashed line). Gene expression differences under each temperature condition were compared using a two-sided Wilcoxon rank sum test (shown above each bracket). Adult tissues at 3°C are underpowered

bioRxiv preprint doi: https://doi.org/10.64898/2025.12.22.696110; this version posted December 25, 2025. The copyright holder for this preprint (which was not certified by peer review) is the author/funder, who has granted bioRxiv a license to display the preprint in perpetuity. It is made available under aCC-BY 4.0 International license.

for detecting significant ASE due to reduced sample number. Expression of *C3* from the BEPA allele decreases at 3°C in adult flank muscle.

(C) Stacked bar plots representing the relative expression levels of *C3* copies from RABS (red) and BEPA (*C3A*, dark blue; *C3B*, medium blue; *C3L*, light blue) determined by mapped Kinnex reads.

ns = not significant, $p > 0.05$, $^*p \leq 0.05$, $^{**}p \leq 0.01$
